# The efficacy of 5-element therapy for senile diabetes with depression: A protocol for a systematic review and meta-analysis

**DOI:** 10.1097/MD.0000000000023622

**Published:** 2020-12-11

**Authors:** Sihan Peng, Xiyu Zhang, Ya Liu, Xiaoxu Fu, Mingyang Zhou, Gang Xu, Chunguang Xie

**Affiliations:** Hospital of Chengdu University of Traditional Chinese Medicine, Chengdu, China/ Chengdu University of Traditional Chinese Medicine, Chengdu, China.

**Keywords:** five-element therapy, meta-analysis, protocol, senile diabetes with depression, systematic review

## Abstract

**Background:**

Senile diabetes with depression is a common and frequently-occurring disease, and it is also a difficult and hot point in domestic and international research. However, the efficiency of combination hypoglycemic agents and antidepressants in the treatment of senile diabetes with depression is poor, and new intervention methods are urgently needed. Research shows the 5-element therapy, as a Chinese traditional non-drug intervention, has definite curative effect on the prevention and treatment of various physical and mental diseases. The purpose of this systematic review and meta-analysis is to evaluate the efficacy of 5-element therapy on senile diabetes with depression.

**Methods:**

The electronic databases including Pubmed, Embase, Cochrane Library, Web of science, Chinese National Knowledge Infrastructure, Wanfang Database, Sino Med,China Biomedical Literature Database will be searched. The time limit for retrieving studies is from establishment to October 2020 for each database. Randomized controlled clinical trials related to 5-element therapy intervention on senile diabetes with depression will be included. Stata V.13.0 and Review manager 5.3 software will be implemented for data synthesis, sensitivity analysis, subgroup analysis, and the assessment of bias risk. We will use the grading of recommendations assessment, development, and evaluation system to assess the quality of evidence.

**Results:**

This study will provide a quantitative and standardized evaluation for the efficacy of 5-element therapy on senile diabetes with depression.

**Conclusion:**

This systematic review and meta-analysis will provide the high-quality evidence to assess whether the 5-element therapy has a positive treatment effect for senile diabetes with depression.

**Registration number:**

INPLASY2020100081.

## 1 Introduction

Senile diabetes refers to diabetes patients aged 60 or above, which has the characteristics of high prevalence rate, insidious onset and great harm etc. Depression is 1 of the common chronic complications of diabetes. About 30% diabetic patients have depressive symptoms, especially the senile diabetes. According to the survey conducted in China in 2008^[1]^ and 2010,^[2]^ the depression rate of diabetes among elderly over 60 years old was above 20%. There is a bidirectional relationship between diabetes and depression, and the risk of depression in diabetic patients is twice that in non-diabetic patients.^[3]^ At the same time, the risk of cardiovascular disease, dementia, death and suicide in diabetes with depression is significantly higher than that in patients with diabetes or depression alone due to the double influence of the 2 diseases.^[4,5]^ Studies have shown that psychological therapy and antidepression drugs are moderately effective in alleviating negative emotions in diabetes with depression, while cognitive behavioral therapy has a more beneficial effect on blood glucose control.^[6,7]^ Traditional Chinese medicine can treat diabetes with depression from aspects of Chinese herbal medicine, acupuncture, traditional exercise and emotional nursing of traditional Chinese medicine,^[8,9]^ which can significantly improve the physical and mental state of senile diabetes with depression as well as their quality of life.^[10]^

The 5-element therapy is an intervention method based on the basic theory of Traditional Chinese medicine, which uses the 5 tones of “Jue, Zhi, Gong, Shang and Yu” to prevent and treat diseases. It combines acoustics and medicine theories together, and it is also the earliest established theoretical system of acoustic medicine with characteristics in the history of world medicine. The Chinese famous doctor Zhu Zhenheng from Yuan dynasty pointed out that “music is also for medicine.” The 5-element therapy emphasizes the unity of body and spirit, pays attention to “the co-cultivation of body and spirit”. Studies have shown that music can stimulate endocrine system of the human body and secrete hormones related to emotion and stress, thus regulating physical and mental health.^[11]^ At present, music therapy as a safe and effective treatment method, has been applied in the rehabilitation of senile dementia,^[12]^ pain relief,^[13]^ schizophrenia treatment,^[14]^ depression symptom relief^[15]^ and other aspects. It is also widely used in clinical surgery, hospice care and other fields.^[16,17]^ In this study, we will assess the efficacy of 5-element therapy in the treatment of senile diabetes with depression, and provide high-quality evidence for the treatment of it.

## 2 Methods

### 2.1 Study registration

This protocol has been registered with International Platform of Registered Systematic Review and Meta-Analysis Protocols on 20 October with registration number INPLASY2020100081.

### 2.2 The inclusion criteria

#### 2.2.1 Types of studies

Randomized controlled clinical trials and quasi-randomized controlled trials will be considered for inclusion in this study. Studies involving non- randomized controlled trials, reviews, animal experiments, case series will be excluded.

#### 2.2.2 Types of participants

Participants diagnosed as diabetes with depression (aged≥60 years)will be included. All participants will regardless of gender and ethnicity.

#### 2.2.3 Types of interventions

This meta-analysis will include the randomized controlled trials of 5-element music therapy regardless of duration and frequency.

#### 2.2.4 Types of comparator(s)/control

Conventional treatment according to relevant guideline, or other forms of Chinese traditional non-drug intervention such as Tai Chi, acupuncture, Tuina, and so on.

#### 2.2.5 Types of outcomes

The primary outcomes of this review are fasting blood glucose (FBG)and 2 hour postprandian blood glucose. The secondary outcomes include the following items: HbA1c, fasting insulin, homeostasis model assessment of insulin resistance, quantitative insulin sensitivity check index.

### 2.3 Collection and analysis of data

#### 2.3.1 Search strategy

The literature search will be performed by using the following databases: Pubmed, Embase, Cochrane Library, Web of science, Chinese National Knowledge Infrastructure, Wanfang Database, Sino Med, China Biomedical Literature Database. Studies published from the inception to October 2020 without language restrictions. The search strategy will be developed by the research team in collaboration with an experienced librarian and checked by a referee according to the Peer Review of Electronic Search Strategy guidelines. The search strategy is listed in Table 1.

**Table 1:** Search tactics for the PUBMED and web of science.

1 Five-element	7 Senile diabetes with depression
2 Five-element therapy	8 Diabetes with depression
3 Five-tone therapy	9 Randomized controlled trial
4 Traditional 5-element therapy	10 Randomized trial
5 Traditional Chinese therapy	11 Controlled clinical trial
6 Senile diabetes	12 Clinical trial

#### 2.3.2 Data selection

**Figure 1:**
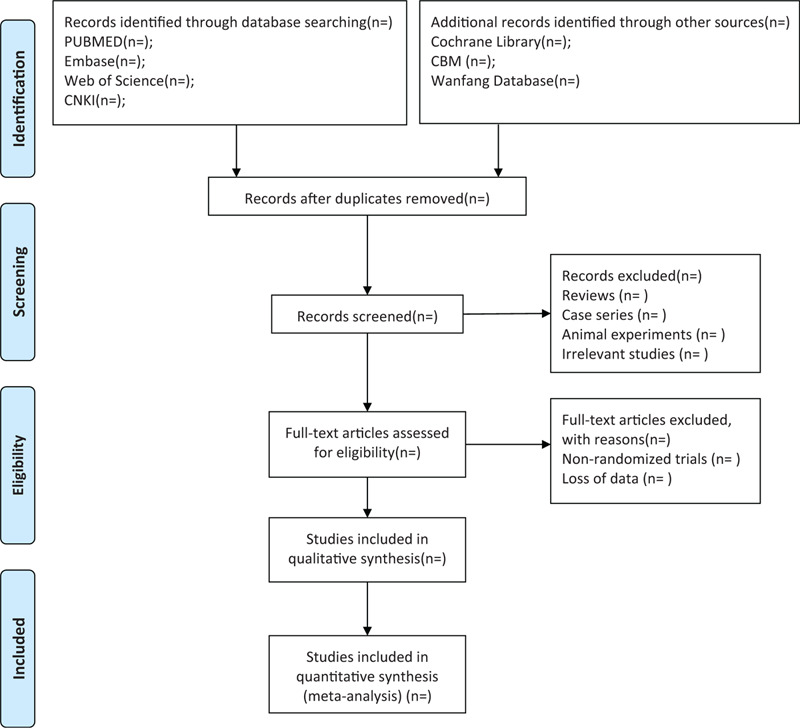
Flow diagram of studies selection process.

All of the authors will be trained with the Preferred Reporting Items for Systematic Reviews and Meta-Analyses and CHSRI from the beginning. Endnote V.X9 will be used to manage literature and remove duplications. The appropriate studies will be searched and screened by 2 independent investigators after reading the titles, abstracts. All repetitions and studies not met the inclusion criteria will be excluded. Then the same investigators will evaluate the full texts, blinded to each other's review. Any disagreements will be settled by discussion between the authors. The process of study selection will be performed according to the Preferred Reporting Items for Systematic Reviews and Meta-Analyses guidelines,^[18]^ and the diagram of this study is shown in Figure 1.

#### 2.3.3 Data extraction

Two independent reviewers will extract data from the included studies based on the eligibility criteria. The following information will be documented from all the included studies: study characteristics, methodological characteristics, participant characteristics, intervention details, outcome indicators for efficacy and safety, follow-up and others. Any disagreements will be rechecked and resolved by consensus. When it's necessary, we will contact with the authors for more related information and clinical data.

#### 2.3.4 Risk of bias assessment

Research design, methods or conduct of study may lead to bias, obscuring the benefit or harm of an intervention. Two investigators will independently assess the risk of bias according to the Cochrane Handbook for Systematic Reviews of Interventions. The following 7 items should be contained, such as random sequence generation, allocation concealment, blinding of participants and personnel, the blindness of outcome assessments, incomplete outcome data, selective outcome reporting, and other biases. The results will be checked repeatedly and the differences will be resolved by further discussion of all reviewers.

### 2.4 Statistical analysis

#### 2.4.1 Assessment of heterogeneity

We will evaluate the clinical heterogeneity at first. Clinical heterogeneity refers to the variation caused by different participants, interventions and different end-point indicators of the study. Then the statistical heterogeneity will be evaluated base on the Cochran *Q* and *I*^2^ test if there's no clinical heterogeneity. The heterogeneity of data will be assessed by calculating the *I*^2^ value. If *I*^2^ < 50%, there's no statistical heterogeneity considered in the study. While *I*^2^ ≥ 50%, significant statistic heterogeneity exists in the trial and the meta-analysis will not be performed.

#### 2.4.2 Synthesis of data

We will utilize the Review Manage software V5.3.0 to analyze all data. And we will calculate the risk ratio (RR) for dichotomous with 95% confidence intervals. For continuous data, mean difference will be included in the meta-analysis. While the outcome variables are measured by different scales, standard mean differences analysis with 95% confidence intervals will be estimated in the meta-analysis as well.

#### 2.4.3 Additional analyses

We will conduct sensitivity analysis, meta-regression and subgroup analysis based on the various study characteristics and samples. For example, the study type, study quality as well as the adjustment for confounders are included in. A brief qualitative analysis of the evidence will be presented in narrative form if data extraction is insufficient or significant differences exist in study methods.

#### 2.4.4 Publication bias

When more than 10 trials are included in the metaanalysis, a funnel plot will be generated to evaluate potential publication bias. And the symmetrical funnel plot indicates low risk, while the asymmetrical funnel plot expresses a high risk of publication bias.

### 2.5 Quality of evidence

The quality of evidence will be assessed by 2 reviewers independently according to the Grading of Recommendations Assessment, Development and Evaluation (GRADE) system. Based on 5 factors (limitation, inaccuracy, inconsistency, indirectness, and publication bias) of GRADE rating standards, the quality of evidence is divided into 4 levels (high, moderate, low, and very low). The GRADE profiler 3.2 will be employed for analysis.

### 2.6 Ethics and dissemination

Ethical approval is not required in this study, because no clinical trials or animal experiments are involved in. Our findings will provide information about the treatment efficacy of 5-element music therapy for depression in senile diabetes patients. The establishment of this study may be published in peer-reviewed journals.

## 3 Discussion

As 1 of the traditional non-drug therapies in China, 5-element therapy has both theories and practical methods. It has outstanding advantages in treating and preventing the diseases, and it also has been widely used in the fields of senile dementia rehabilitation, schizophrenia treatment, clinical surgery as well as hospice care and so on. However, there is no systematic and comprehensive meta-analysis on the efficacy of 5-element therapy for senile diabetes with depression. Therefore, we intend to conduct meta-analysis of the efficacy of 5-element therapy in the treatment of senile diabetes with depression, in order to provide high-quality evidence and guidance for clinicians and scientific researchers.

## Author contributions

**Conceptualization**: Sihan Peng

**Data collection:** Sihan Peng, Xiyu Zhang, Ya Liu

**Statistical analysis**: Xiaoxu Fu

**Supervision**: Mingyang Zhou

**Software**: Gang Xu

**Writing – original draft**: Sihan Peng

**Writing – review & editing**: Chunguang Xie
